# Plant functional trait diversity regulates the nonlinear response of productivity to regional climate change in Tibetan alpine grasslands

**DOI:** 10.1038/srep35649

**Published:** 2016-10-19

**Authors:** Jianshuang Wu, Susanne Wurst, Xianzhou Zhang

**Affiliations:** 1 Lhasa National Ecological Research Station, Key Laboratory of Ecosystem Network Observation and Modelling, Institute of Geographic Sciences and Natural Resources Research, Chinese Academy of Sciences, 100101 Beijing, China; 2 Functional Biodiversity, Dahlem Center of Plant Sciences, Free University of Berlin, 14195 Berlin, Germany

## Abstract

The biodiversity-productivity relationship is still under debate for alpine grasslands on the Tibetan Plateau. We know little about direct and indirect effects of biotic and abiotic drivers on this relationship, especially in regard to plant functional trait diversity. Here, we examine how aboveground net primary productivity (ANPP) and precipitation use efficiency (PUE) respond to climate, soil and community structure across alpine grasslands on the Northern Tibetan Plateau. We found that both ANPP and PUE showed nonlinear patterns along water availability and site altitude variation, which together accounted for 80.3% and 68.8% of variation in ANPP and PUE, respectively, by optimal generalized additive models. Functional trait divergence (FTD) and community weighted mean (CWM) of plant functional traits were as important as plant species diversity (PSD) for explaining the nonlinear productivity-climate relationship. These findings were confirmed by results from principal component analyses and structural equation models. We also found that FTD was negatively correlated with PSD across different alpine grasslands. Our results implicate: first, the combinatorial influences of temperature and precipitation gradients are important for predicting alpine grassland dynamics; second, the convergence and divergence of plant functional traits may have the potential to elucidate the effect of plant diversity on ecosystem functionality.

Biodiversity conservation is one of the most important issues that closely link human welfare and ecosystem functions in a rapidly changing world^1^,^2^,^3^,^4^. The current studies on the relationship between biodiversity and ecosystem functions are increasingly moving their focus from species diversity indices to trait-based approaches^5^,^6^,^7^,^8^,^9^,^10^. Interactions between biodiversity and ecosystem functions are argued not necessary to be linear or unimodal across different scales. Therefore, the relationship between biodiversity and ecosystem functionality calls for causal network analyses rather than simply bivariate analyses to understand mechanisms. However, it is still unclear and under debates how biodiversity changes affect community productivity on alpine grasslands.

On the Tibetan Plateau, alpine grasslands are sensitive and vulnerable to both natural and anthropogenic disturbances^11^. Degradation of alpine grasslands is mainly attributed to climate warming and overgrazing there^12^,^13^,^14^,^15^,^16^. For example, Piao *et al*.^17^ found that the spatial patterns of vegetation green-up and its change in response to warming are altitude dependent. As these pastures are an important ecological security by protecting the headwaters of major rivers in Asia, their ongoing degradation likely threatens the livelihood of local residents and their distinctively nomadic culture^11^,^18^, but may also have wider impacts on water security in East China and South-Asia. Assumed to effective to recover degraded pastures, metal fences were built on this plateau to control grazing by livestock^12^,^18^. However, the magnitude and direction of fencing effects on plant communities and soil properties are still under debates^19^,^20^,^21^,^22^,^23^,^24^,^25^. The questions whether a general biodiversity-productivity relationship exists for Tibetan alpine grasslands and how this relationship is affected by climatic factors are still under discussion.

A positive linear relationship between species richness and productivity has been reported for Tibetan alpine grasslands^26^,^27^. However, no relation was found between the corresponding residuals of species richness and productivity after environmental and climatic influences were removed. Ma, *et al*.^26^ and Wang, *et al*.^27^ argued that plant species richness can weakly affect community productivity across Tibetan alpine grasslands. In fact, intrinsic community properties, for instance, community assembly of plant functional groups, can be as important as extrinsic abiotic divers in explaining spatial and temporal variabilities of grassland productivity across the Tibetan Plateau^19^,^28^,^29^,^30^. Current findings are mainly deducted from bivariate analyses, and insight studies on direct and indirect interactions between potential biotic and abiotic are still rare.

Plant functional trait diversity is useful to address questions of plant performance, community assembly and ecosystem functions along climate, resources and disturbance gradients^10^,^31^,^32^,^33^,^34^. For example, changes in plant trait composition in response to grazing vs. mowing disturbance can be distinguished and predicted by functional trait means and divergence^35^,^36^. Maintaining and enhancing functional trait diversity might be vital to buffer the negative effects of climate change and human activities on ecosystem multifunctionality^34^,^37^, especially for dry and alpine grasslands on our planet. He, *et al*.^38^ explored the general bivariate relationship between plant leaf traits of 74 species sampled from 49 sites on the Qinghai-Tibetan Plateau, but they did not link plant functional traits with ecosystem functionality.

Precipitation use efficiency (PUE) is a widely used proxy for the sensitivity of grassland productivity in response to precipitation^39^,^40^,^41^. Hu, *et al*.^42^ and Yang, *et al*.^43^ examined the spatial PUE pattern across different alpine grassland types, explained the potential influences of climatic variables and soil properties, but did not examine the direct and indirect influences of plant functional trait diversity. Current studies that follow trait-based approaches are limitedly conducted in the humid meadows in the eastern Tibetan plateau^28^,^44^,^45^,^46^ and rarely in the arid steppe and desert-steppe zones in the northwestern region^47^. At large spatial scales, from both field observations and remote sensing modelling, scientists have confirmed that Tibetan alpine grasslands are very sensitive to changes in precipitation^28^,^29^,^48^,^49^,^50^. However, gaps in understanding the interactions between biodiversity, functionality and precipitation changes still remain.

In this paper, we concretely aim (1) to examine differences in biodiversity and functionality among zonal alpine grassland types, especially focusing on for functional trait diversity indices; (2) to clarify the spatial patterns of biodiversity and functionality along geographical, edaphic and climatic gradients at a regional scale; (3) to figure out direct and indirect links between biodiversity and functionality in response to the regional water availability gradient, an indicator of habitat moisture. In addition, we also want to find out whether plant functional trait diversity indices are more effective than the traditional plant species diversity (PSD) indices, such as richness, dominance and evenness, in explaining the sensitivity of alpine grassland productivity to regional climatic gradients across the Tibetan Plateau.

## Methods

### Study area and sampling

#### Study area

**Figure 1:**
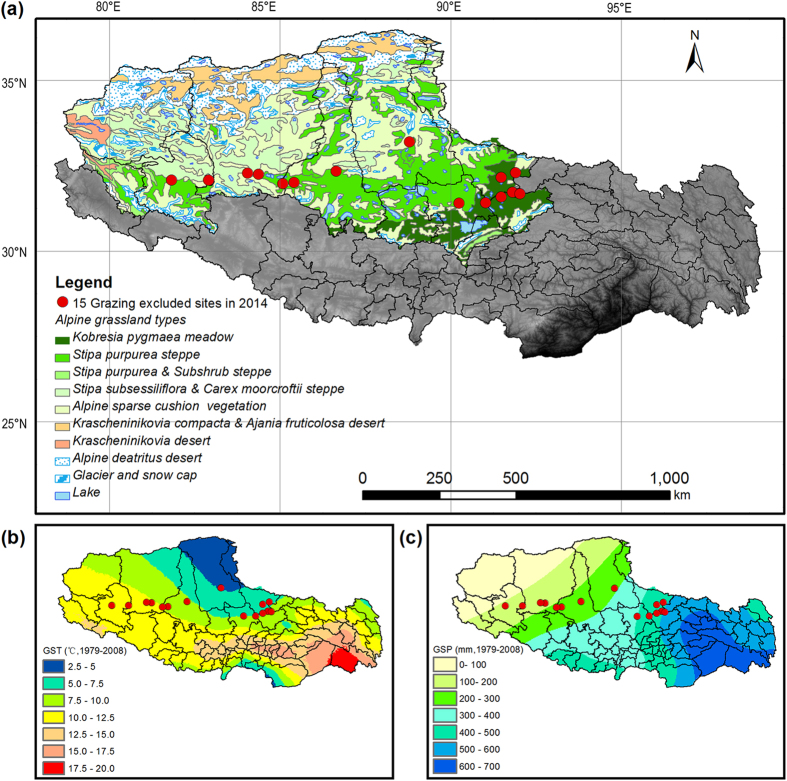
Study region, climate and sites sampled across the Northern Tibetan Plateau. (**a**)-regional context of alpine grassland types (AGTs) showed site locations. (**b**)-mean annual temperature (MAT) and (**c**)-mean annual precipitation (MAP) over the Tibetan Autonomous Region, China, indicating the climatic conditions of our sampling sites. ArcGIS10.2 was used to create these maps. Climatic data ranges from 1979 to 2008 and daily weather records are available from the China Meteorological Data Sharing Service System.

We sampled 75 plots at 15 sites in total during the peak-growing season of 2014, with five sites for each grassland type, westwardly across humid alpine meadow (AM) dominated by *Kobresia pygmaea*, semi-arid alpine steppe (AS) dominated by *Stipa purpurea*, and arid alpine desert-steppe (ADS) co-dominated by *S. purpurea* and *S. glareosa* on the Northern Tibetan Plateau (29°53′–36°32′N; 78°41′–92°16′E) (Fig. 1 and Supplementary Table S1). Plant growing season (GSP) there generally starts in early May and ends in late September when up to 85% of annual precipitation happens and mean daily temperature is always over 5.0 °C^51^. To minimize disturbances by domestic livestock and wild herbivores, sampling was conducted at fenced pastures. Thus, the peaking aboveground biomass can be accepted as aboveground net primary productivity (ANPP).

#### Sampling design

At each site, we established a sub-enclosure of 200 m × 200 m in size for measuring species composition, leaf traits and aboveground biomass. Five 50 cm × 50 cm plots were independently arranged at 20-m intervals along a random transect line. We identified plant species occurring, measured general leaf height (GLH, cm), and estimated percentage cover for each species within each plot. Aboveground biomass was harvested, sorted by species and stored in paper bags. Plant samples were dried at 65 °C for 48 h and weighted to 0.001 g so that rare species could also be identified. Species frequency was additionally sampled using thirty 0.1 m^2^ random circles at each site. We also measured specific leaf area (SLA, cm^2^g^−1^) and leaf mass fraction (LMF, %) as plant functional traits^52^.

### Data management

#### Species and trait diversity indices

We firstly calculated species relative dominance based on percentage cover, leaf height and frequency measured at each site as did Wu, *et al*.^22^. Then we defined species richness (*SR*) as the total number of species at each quadrat (Plot_SR). Next, we calculated Shannon index of diversity (Shannon), Simpson index of diversity (Simpson), and Pielou evenness index (Pielou)^53^. Plot-SR, Shannon, Simpson and Pielou were grouped as plant species diversity (PSD) indices.

To compare community trait composition of GLH, SLA and LMF, we calculated community weighted mean (CWM) values for each quadrat following the equation of Violle, *et al*.^10^ and Ricotta and Moretti^54^.


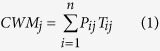


where the *P*_*ij*_ is the relative dominance (cover percent) of the species *i* in the quadrat *j, T*_*ij*_ is the mean trait value of the species i in the quadrat *j*, and *CWM*_*j*_ is the community-weighted trait of the quadrat *j*.

We also followed the distance-based framework of Laliberte and Legendre^53^ to quantify the variance of community trait distribution. Functional trait divergence (FTD) that reflects niche complementarity (overlaps/divergence) was calculated for plant GLH, SLA and LMF, respectively.


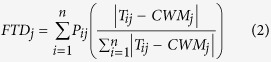


where the *P*_*ij*_ is the relative dominance of the species *i* in the quadrat *j, T*_*ij*_ is the mean trait value of the species *i* in the quadrat *j*, and *FTD*_*j*_ is the community-weighted trait of the quadrat *j*.

#### Climatic variables and topsoil nutrients

We downloaded daily records of precipitation and temperature from the China Meteorological Data Sharing Service System (http://cdc.nmic.cn/home.do). As low-temperature stress is common over the Tibetan Plateau, plants generally start to grow when it is warm. In addition to GSP, accumulated temperature when daily mean is over 5 °C (AccT) also accumulated^27^. GSP and AccT in 2014 were firstly compiled from daily climatic raster surfaces that were interpolated using ANUSLPIN 4.3^55^, and then extracted for each site in ArcGIS 10.2 (ERSI, Redlands, CA, USA). Finally, we accepted the ratio of GSP/AccT to describe the habitat moisture index (HMI = GSP/AccT) as did Wang, *et al*.^27^. Soil variables, including soil organic carbon (SOC) and total nitrogen (STN) were extracted from Wu, *et al*.^51^ and Li, *et al*.^56^.

### Statistical analyses

#### Geospatial responses of alpine grasslands to regional abiotic gradients

As plant communities are not necessary to linearly respond to environmental changes^57^, generalized additive models (GAM) were induced to cope with problems such as collinearity and non-linearity between responsible and explanatory variables^58^ using the *mgcv* package in R^59^. Here, abiotic explanatory variables include site geographical locations (longitude, latitude, and altitude), climatic factors (GSP, AccT and HMI) and soil properties (SOC, STN and C: N ratio). The response variables were ANPP and PUE at the plot level. We followed a backward selection approach with Akaike Information Criterion (AIC) and Bayesian Information Criteria (BIC) to find the optimal models out^58^,^60^ (Supplementary Figures S1 and S2 and Tables S2 and S3).

#### Functional and structural differences between alpine grassland types

Functional and structural differences among zonal alpine grassland types were initially examined by one-way analysis of variance (ANOVA) with Tukey HSD test (*P* < 0.05) (Supplementary Figure S3). Next, we conducted principal component analyses (PCA) to examine functional and structural differences of communities surveyed, firstly did separately for PSD, CWM and FTD, and then pooled them together. We used the PCA coordinates with an eigenvalue >1 to identify the plant strategy along which trait or species diversity indices co-vary across communities.

#### Direct and indirect effects of water availability on ecosystem function

**Figure 2:**
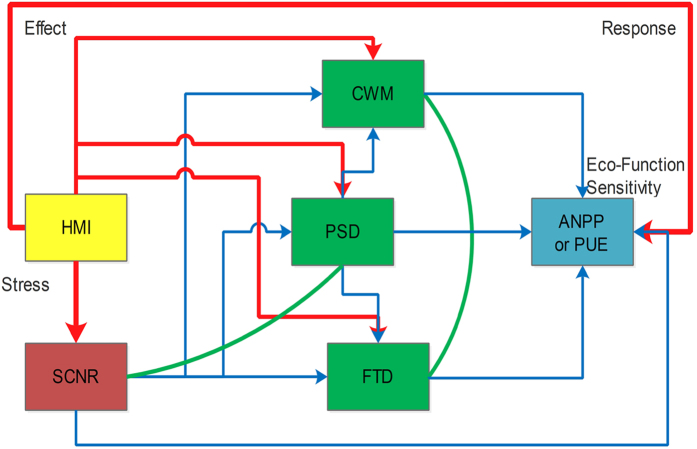
Hypothetical interrelations among climate, soil, community structure, and ecosystem function (sensitivity) of alpine grasslands on the Northern Tibetan Plateau. Red lines are for direct impacts, blue lines for indirect impacts, and green lines for covariations. Habitat moisture index (HMI) equals the ratio of growing season precipitation (GSP) to accumulated temperature when daily mean is over 5 °C (AccT). Soil C: N ratio (SCNR) was also considered in this study. Plant species diversity (PSD) includes the indices of richness, Shannon, Simpson, Pielou evenness at the plot level. Community weighted means (CWM) and functional trait divergence (FTD) were calculated from general leaf height (GLH, cm), specific leaf area (SLA, cm^2^ g^−1^) and leaf mass fraction (LMF, %).

For the biodiversity-functionality relationship, the causal network analysis is likely more effective to clarify direct and indirect interrelations than bivariate regressions or analyses of variances^59^. Plant functional trait diversity may provide more mechanistic understandings than species diversity indices when relates to ecosystem functionality^10^. We conducted structural equation model (SEM) using the lavaan package^61^ in Rstudio^59^ to explore interrelations among climate, soil, community structure, and ecosystem function (sensitivity) as hypothesized in Fig. 2.

## Results

### Geospatial patterns of ANPP and PUE across studied communities

**Figure 3:**
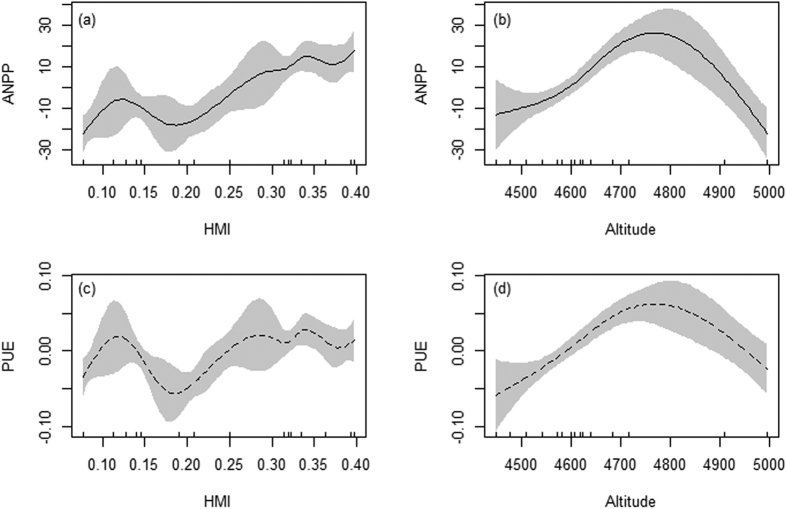
The estimated smoothers for ANPP (**a**,**b**, solid lines) and PUE (**c**,**d**, dashed lines), respectively, obtained by the optimal generalized additive models (GAMs) that include habitat moisture index (HMI, **a**,**c**) and site altitude (**b**,**d**) as explanatory variables. The grey areas show the 95% point-wise confidence bands.

Due to collinearity between explanatory variables (Supplementary Figures S1 and S2), soil parameters were excluded from the optimal models (Supplementary Tables S2 and S3). The smoothers of HMI and site altitude were significant, entered the optimal models, and together explained 80.3% of the variation in ANPP and 68.8% of variation in PUE (Table 1), respectively. The patterns of ANPP and PUE are similar, showing clear increasing trends along the HMI gradient from approximately 0.18 mm °C^−1^ to the most humid sites 0.4 mm °C^−1^, and unimodal patterns at the dry end of the HMI gradient, lower than 0.18 mm °C^−1^ (Fig. 3a,c). Along the site altitudinal gradient, both ANPP and PUE show unimodal patterns with peaks at approximately 4800 m (Fig. 3b,d).

### Grassland functional structure based on plant species and leaf traits

**Figure 4:**
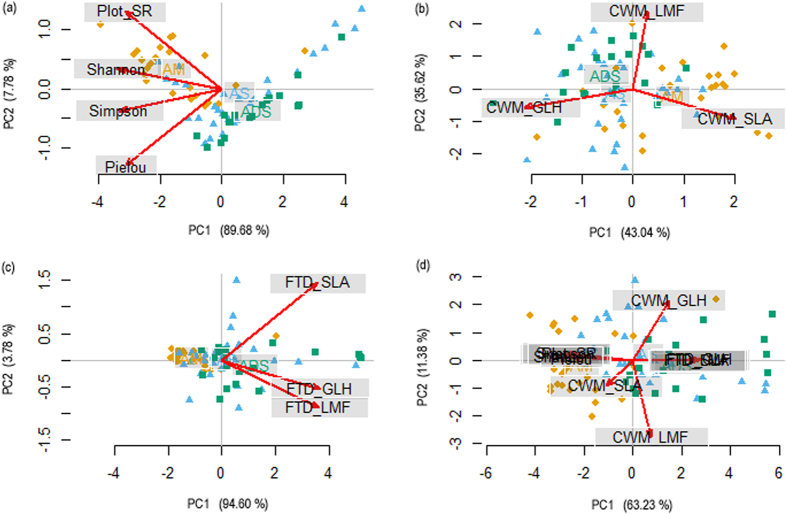
Principal component analyses (PCA). (**a**)-based on plant species diversity (PSD) that includes species richness at the plot level (Plot_SR), Shannon diversity index (Shannon), Simpson dominance index (Simpson) and Pielou evenness index (Pielou); (**b**)-based on community weight means (CWM) of general leaf height (CWM_GLH), leaf mass fraction (CWM_LMF) and specific leaf area (CWM_SLA); (**c**)-based on functional trait divergence (FTD) of general leaf height (FTD_GLH), leaf mass fraction (FTD_LMF) and specific leaf area (FTD_SLA); and (**d**)-with all potential explanatory variables being pooled together. Yellow diamonds, blue triangles and green squares represent alpine meadow (AM), steppe (AS) and desert-steppe (ADS), respectively. For each component we indicate the percentage of variance explained. See Supplementary Figure S1 for correlations between PSD, CWM and FTD values, Figure S3 for site locations within each alpine grassland type (AGTs) and climatic background, and Figure S4 for variable comparisons among the three AGTs.

The biodiversity indices that were separately grouped into PSD, CWM and FTD, showed different explanatory powers for variance of community structure in this study. The PCA results indicated that the explanatory power of PSD is comparable to the FTD group, the former accounting for 89.68% (Fig. 4a) vs. the latter for 94.60% (Fig. 4c) of the total variance explained by the first component. The CWM group can account for 78.66% of the total variance, with the first component (43.04%) highly correlated with LMF and the second component (35.62%) highly correlated with GLH and SLA (Fig. 4b). When all community structural predictors were pooled together, the first PCA components (63.23% of the variance) separated communities according to the PSD and FTD values (Fig. 4d, with PSD being negatively correlated to FTD, to see Supplementary Figure S1) while the second PCA component (11.38% of the variance) discriminated communities according to the CWM indices.

### The causal networks for ANPP and PUE across studied communities

**Figure 5:**
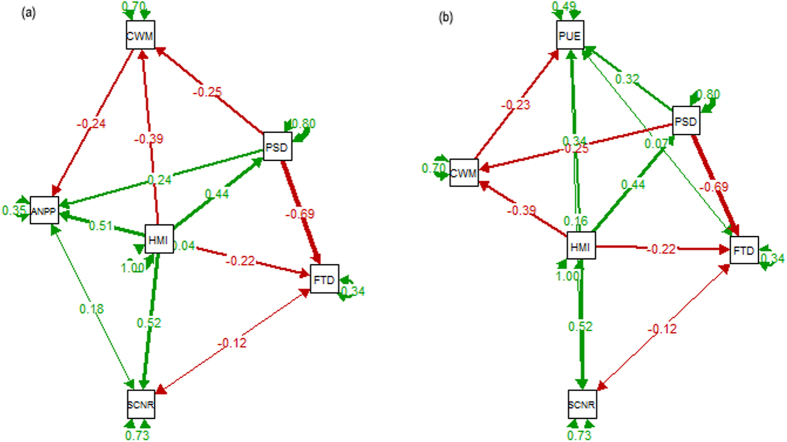
Structural equation models examining effects of environmental factors and biodiversity components on ANPP (**a**) and PUE (**b**), respectively, at the regional scale across zonal alpine grassland types on the Northern Tibetan Plateau. Green and red arrows indicate significant positive and negative effects, respectively. Line width illustrates path strength. Values associated with path arrows represent standardized path coefficients. Values near to the round arrows show the residuals. Non-significant paths and variables were eliminated from the final SEMs. Results of model fitting: (**a**) χ^2^ = 1.513, *P* = 0.679; (**b**) χ^2^ = 1.513, *P* = 0.679 (Note: higher *P*-values associated with χ^2^ tests indicate better fitting to data. See Supplementary Table S3 for summaries of the full SEMs with non-significant relationships included for ANPP and PUE, respectively.

The influence of HMI on ANPP was mainly mediated through PSD and CWM of plant functional traits. HMI directly (*β* = 0.51, standardized coefficient), and indirectly through CWM (*β* = −0.24, standardized coefficient) and PSD (*β* = 0.24, standardized coefficient), impacted ANPP (Fig. 5a). The indirect influences of FTD of plant functional traits and soil properties (SCNR) on ANPP were not significant (Supplementary Table S4). SCNR was controlled by HMI (*β* = 0.52, standardized coefficient) while FTD was mainly determined by PSD (*β* = −0.69, standardized coefficient). The influence of HMI on PUE was significantly mediated by PSD (*β* = 0.32, standardized coefficient) and CWM (*β* = −0.23, standardized coefficient) (Fig. 5b). The indirect influence of HMI through PSD on PUE was approximately equal in magnitude to the direct influence of HMI (*β* = 0.34, standardized coefficient). The FTD of plant traits and soil properties (SCNR) were no significant impacts on PUE (Supplementary Table S4). The strongest relationship observed in the SEM analyses was between PSD and FTD (*β* = −0.69, standardized coefficient).

## Discussion

### Geospatial nonlinear patterns of productivity and collinearity between abiotic factors

Alpine grasslands are generally believed to be sensitive to climate change. Unfortunately, the question how community structure under ongoing climate change affects ecosystem functionality is still poorly understood. To mechanistically understand this question, we have examined the spatial patterns of productivity-biodiversity relationship within an effect-response framework across three zonal alpine grasslands types on the Northern Tibetan Plateau. Our results have shown that, at the regional scale, both ANPP and PUE (termed as the ratio of ANPP to GSP) nonlinearly respond to the increasing water availability (HMI) gradient at the regional scale (Fig. 3). Importantly, the unimodal ANPP-altitude relationship at such large geospatial scale was consistent with the finding of Wang, *et al*.^27^ at a local scale, along a mountain slope in the central Tibetan Plateau. Therefore, our results supported hypothesis that ecological processes are not necessary to linearly respond to climate change either^62^,^63^,^64^.

The responses of alpine grassland communities subject to abiotic and biotic factors have been explored by a few studies based on bi-variate analyses^29^,^42^,^43^. But the collinearity between explanatory variables and the nonlinear response patterns have rarely documented. For example, Yang, *et al*.^43^ reported a unimodal pattern of PUE along mean annual precipitation (MAP) across alpine steppe and meadow communities on the Tibetan Plateau, and attributed it to the linear bivariate relationships of PUE with species richness, soil organic carbon and soil silt content. As soil nutrients were highly correlated with HMI (Supplementary Figure S2), in this study soil explanatory variables were excluded from the optimal GAMs. Soil C: N was also confirmed to have no significant impacts on either ANPP or PUE in the full SEMs (Supplementary Figure S4 and Table S4). This may be attributed to the historical co-evolution of climate, vegetation and soils, because soil development in alpine biomes and mountain regions is mainly controlled by climatic condition there^65^. Therefore, our findings on the collinearity between soil nutrients and climatic factors implied that the dynamics of community structure and ecosystem functionality might be weakly influenced by soil nutrients. This can also explain why no relation exists between corresponding residuals of species richness and productivity after removing environmental influences^26^,^27^.

Vegetation at high altitude is generally believed to be more sensitive to global changes than other biomes because of the extreme habitat conditions^65^,^66^, for instance, stress due to low temperatures and poor soil nutrient availability affect plant performance and reproduction. In addition, we found the optimal GAMs that included HMI and site altitude (adj. R^2^ = 0.803, AIC = 559.77 for ANPP; adj. R^2^ = 0.688 AIC = −327.74 for PUE) were a bit better that the sub-optimal models that included GSP and site altitude (adj. R^2^ = 0.798, AIC = 561.71 for ANPP; adj. R^2^ = 0.676, AIC = −325.40 for PUE) (Supplementary Tables S2 and S3), although a few studies have argued that precipitation tends to be more important than temperature for alpine grassland productivity^29^,^67^. Our results support that alpine grassland dynamics closely related the wet/dry condition and influenced by the combination of temperature and precipitation^27^,^50^,^68^. The combination of temperature and precipitation varies along an increasing altitudinal gradient to affect community assembly and ecosystem productivity^27^. Therefore, the unimodal patterns of ANPP and PUE at the dry end of HMI gradient in this study might be modified by site altitude differences, because we have examined unimodal patterns of ANPP and PUE against site altitude at the regional scale. Therefore, our results indicate that elevation dependency of climate changes, especially in the combination of temperature and precipitation, should be also clarified for predicting vegetation dynamic in montane regions^27^,^69^.

### Ecosystem causal networks and explanations for ANPP and PUE from biotic factors

Previous studies showed that drier places tend to have lower ANPP because of sparser community, higher evaporation potential, and higher tolerance to drought stress^39^,^41^,^70^,^71^. In general, we found that PUE showed an increasing trend along the HMI gradient from approximately 0.18 mm °C^−1^ to the most humid sites 0.4 mm °C^−1^ (Fig. 3c), being partly consistent with the positive linear relationship between PUE and mean annual precipitation (MAP) reported by Hu, *et al*.^42^. However, a unimodal pattern between PUE and HMI was found at places with HMI lower than 0.18 mm °C^−1^ (Fig. 3c), being partly consistent with findings of Yang, *et al*.^43^. In addition to the influence of site altitudes, the shifts of ANPP and PUE patterns, from unimodal to approximately linear, may also be attributed to changes in species assembly and trait divergence. Community assembly of plant functional groups have been confirmed by bivariate regressions to be as important as precipitation in controlling and explaining the spatial variation in ANPP^28^. In this study, we also found high correlations of ANPP (PUE) with PSD and FTD indices (Supplementary Figure S1). In addition, comparisons of FTD among alpine grassland types showed that plant functional traits were more convergent with lower averages and smaller variations in humid meadows than semi-arid steppes and arid desert-steppes (Supplementary Figure S3). This may also contribute to the unimodal patterns of ANPP and PUE at the end of HMI.

Interior changes of community assembly, including species composition, plant functional groups and traits, can consequently regulate ecosystem function and sensitivity in response to exterior driver for predicting potential responses to regional environmental changes^40^,^72^,^73^,^74^,^75^,^76^. Surprisingly, we found that PSD had similar importance as FTD in explaining the variance of alpine grassland communities from the PCA results (Fig. 4a,c) and that PSD had a significant impact negative impact on FTD with the highest standardized coefficient (*β* = −0.69) in the final SEMs (Fig. 5). This may be explained by the environmental filtering theory that community assembly is not a random process but based on the filtering of environmental factors on plant functional traits^31^,^77^,^78^. The indirect impact of HMI through PSD was thereby found as strong as the indirect impact through CWM (having same standardized coefficients, Fig. 5a). FTD had no significant effect on ANPP or PUE due to covariations with PSD in the SEM analyses (Fig. 5 and Table 2). These findings indicate that alpine plants and communities have been adapted to local abiotic conditions because of environmental filtering effects on plant trait evolution. It should be clarified that both CWM and FTD, in addition to plant trait values, are also determined by species relative dominance as shown in their definitions. The productivity sensitivity to climate changes at the community level can also be modified by changes in plant relative dominance. Therefore, we suggest that the flexibility of plant functional traits within and across species as well as their dominance changes in response to climate change and human disturbance should be seriously considered in long-term and large-scale studies on ecosystem functionality.

In this study, we explored the patterns of alpine grassland productivity (ANPP) and its sensitivity (PUE) in response to regional climate changes, and examined both strength and direction of biotic and abiotic drivers within the effect-response framework. First, the nonlinearity of ANPP and PUE in response to abiotic variables was detected by general additive models, in which HMI and altitude entered and together accounted for 80.3% and 68.8% of the variation in ANPP and PUE respectively. This result highlights the combinational influences of climate warming and precipitaoitin fluctuation on alpine grassland dynamics. Because the combination of temperature and precipitation always varies along increasing altitudinal gradients, the elevation-dependency should also pay more attention in modelling alpine grassland responses to climate change in this plateau and other mountainous regions. Second, both principal component analyses and structural equation model confirmed that functional trait diversity, including community weighted means and functional trait divergence, is as important as plant species diversity for explaining the nonlinear relationship between climate changes and ecosystem productivity in alpine grasslands on the Northern Tibetan Plateau. These findings are likely due to the collinearitiy of abiotic explanatory factors with climate gradients. On the other hand, due to the environmental filtering effects, plants have been adapted to alpine habitat conditions with highly differential functional traits. Trait flexibility within and across species and changes in plant relative dominance should be examined for long-term studies on the biodiversity-ecosystem functionality relationship.

## Additional Information

**How to cite this article**: Wu, J. *et al*. Plant functional trait diversity regulates the nonlinear response of productivity to regional climate change in Tibetan alpine grasslands. *Sci. Rep.* **6**, 35649; doi: 10.1038/srep35649 (2016).

## Supplementary Material

Supplementary Information

## Figures and Tables

**Table 1:** The summary of the optimal generalized additive models (GAMs) with habitat moisture index (HMI) and site altitude (Alt.) as explanatory variables.

	Predictors	Est. *df*	Est. rank	*F*	*P*	Adj.R^2^
ANPP	HMI	6.605	7.255	13.770	<0.001	0.803
	Alt.	3.433	3.926	18.830	<0.001	
PUE	HMI	7.246	7.886	5.710	<0.001	0.688
	Alt.	2.936	3.348	13.060	<0.001	

Summaries for the non-optimal and sub-optimal GAMs can be found in the Supplementary Table S2 and Table S3 with values of Adjusted R-square, Akaike Information Criterion (AIC) and Bayesian Information Criteria (BIC) provided.

**Table 2:** Summaries of the optimal structure equation models (SEMs) for aboveground net primary productivity (ANPP) and precipitation use efficiency (PUE), respectively.

SEM for ANPP			SEM for PUE		
Regressions:		Estimate	*P* (>|z|)	Estimate	*P* (>|z|)
ANPP~	HMI	0.992	0.000	0.144	0.000
	PSD	0.483	0.002	0.138	0.001
	CWM	−0.992	0.003	−0.200	0.018
CWM~	HMI	−0.184	0.003	−0.184	0.000
	PSD	−0.124	0.018	−0.124	0.018
FTD~	HMI	−0.214	0.003	−0.214	0.003
	PSD	−0.691	0.000	−0.691	0.000
PSD~	HMI	0.429	0.000	0.429	0.000
SCNR~	HMI	0.616	0.000	0.616	0.000
Covariations					
ANPP~~	FTD	0.000	0.715	0.000	0.568
ANPP~~	SNCR	0.002	0.128	0.001	0.165
FTD~~	SCNR	−0.001	0.312	−0.001	0.312
R-square	ANPP	0.661	R-square	PUE	0.507
	CWM	0.302		CWM	0.302
	FTD	0.669		FTD	0.664
	PSD	0.195		PSD	0.195
	SCNR	0.270		SCNR	0.270
Fitness	AIC	−934.834	Fitness	AIC	−1140.584
	BIC	−895.437		BIC	−1101.187

Estimate and significance (*P* value) for regressions (~) and covariations (~~) were shown.

R-square values for each dependent variable were also estimated. Habitat moisture index (HMI) that equals the ratio of growing season precipitation (GSP) to accumulated temperature when daily mean is over 5 °C (AccT). Soil C: N ratio (SCNR) combines carbon and nitrogen in top soils. Plant species diversity (PSD) includes indices of richness, Shannon, Simpson, Pielou evenness at the plot level. Community weighted mean (CWM) and functional trait divergence (FTD) were calculated from general leaf height (GLH), specific leaf area (SLA), and leaf mass fraction (LMF). Values of the Akaike Information Criterion (AIC) and Bayesian Information Criteria (BIC) for each model were provided. See Supplementary Table S4 for methods for rescaling data.
