# Supplementary material for: Plant functional trait diversity regulates the nonlinear response of productivity to regional climate change in Tibetan alpine grasslands
Source: Sci Rep. 2016 Oct 19;6:35649. doi: 10.1038/srep35649 (PMC5069490; doi:10.1038/srep35649)
Supplement: Supplementary Information [file srep35649-s1.pdf]

Plant functional trait diversity regulates the nonlinear response of productivity to regional climate change in Tibetan alpine grasslands

Jianshuang Wu [1*+,2], Susanne Wurst [2], Xianzhou Zhang[1*+]
[1]Lhasa National Ecological Research Station, Key Laboratory of Ecosystem Network Observation and Modelling, Institute of Geographic Sciences and Natural Resources Research, Chinese Academy of Sciences, 100101 Beijing, China
[2]Functional Biodiversity, Dahlem Center of Plant Sciences, Free University of Berlin, 14195 Berlin, Germany
∗ Corresponding: wujs.07s@igsnrr.ac.cn or zhangxz@igsnrr.ac.cn
+ these authors contributed equally to this work.

Supplementary Figures and Tables

Table S1 Alpine grassland types (AGTs), climate and locations of the 15 sites in this study. AGTs include alpine meadow (AM), steppe (AS) and desert-steppe (ADS). Climate factors are growing season precipitation (GSP), > 5°C accumulated temperature (AccT) and habitat moisture index (HMI, = GSP/AccT) in 2014. Location information are geographical coordinates for each sites, including longitude, latitude and altitude.

Site	AGT	GSP, mm	AccT °C	HMI mm °C^{-1}	Longitude °E	Latitude °N	Altitude m, a.s.l
01	AM	395.97	1202.51	0.3228	91.80929	31.72329	4684
02	AM	400.37	1092.02	0.3628	91.48213	32.16452	4618
03	AM	449.11	1132.94	0.3934	91.90795	32.30287	4715
04	AM	394.34	1251.05	0.3139	92.02544	31.67625	4623
05	AM	394.35	1187.95	0.3352	91.48640	31.59342	4573
06	AS	380.25	1194.58	0.3192	91.02171	31.42341	4582
07	AS	305.19	1201.90	0.2507	90.24560	31.41245	4640
08	AS	323.36	807.42	0.3976	88.79698	33.20569	4995
09	AS	267.88	1281.21	0.2084	86.65187	32.33941	4542
10	AS	289.91	1515.46	0.1900	85.40892	32.01195	4909
11	ADS	231.14	1567.38	0.1457	85.07968	31.97696	4600
12	ADS	226.86	1601.54	0.1403	84.37028	32.25004	4510
13	ADS	181.33	1631.67	0.1129	84.05401	32.28900	4448
14	ADS	218.69	1720.81	0.1282	82.91047	32.08037	4477
15	ADS	135.15	1771.52	0.0767	81.82106	32.08144	4607

General Additive (Mixed) Models (GAMs) Selection

Specifically, the least significant term will be dropped until the AIC value of the candidate model is the lowest and all explanatory terms are significant. Moreover, it is perhaps better not to use significantly correlated factors as predictors in the same model. We firstly categorized the nine abiotic potential explanatory variables into three relatively independent groups to build geographical, climatic and edaphic GAM, respectively. For each group, we build model with all potential explanatory variables unless the pairwise correlation is reasonably high with a Pearson correlation coefficient of > 0.6 (or < -0.6). Then we tried to build mixed models with geographical, climatic and edaphic predictors together for all biotic responsible variables, respectively. Once the optimal GAM was selected out, we replaced the most significant predictor with its highly correlated term, and then examined the performance of the potentially alternative model.

Figure S1 Correlation-boxplot- matrix of community functions with/between biotic variables. Aboveground net primary productivity (ANPP) and precipitation use efficiency (PUE) described the community functions. Biotic variables included plot species richness (Plot-SR), Shannon Index, Simpson index, Pielou evenness, and community weighed means (CWM) and functional trait divergence (FTD) of general leaf height (GLH), leaf mass fraction (LMF) and specific leaf area (SLA). The upper panels show the pair-wise scatter plots with smoothers to visualize the patterns, the lower panels contain the Pearson correlation coefficients that is proportional to its estimated value and the diagonal panels show the box plot and the name of the variables which is on the *x*-axis below and above it, and on the *y*-axis left and right of it. The code was taken from the pairs help file in RStudio.

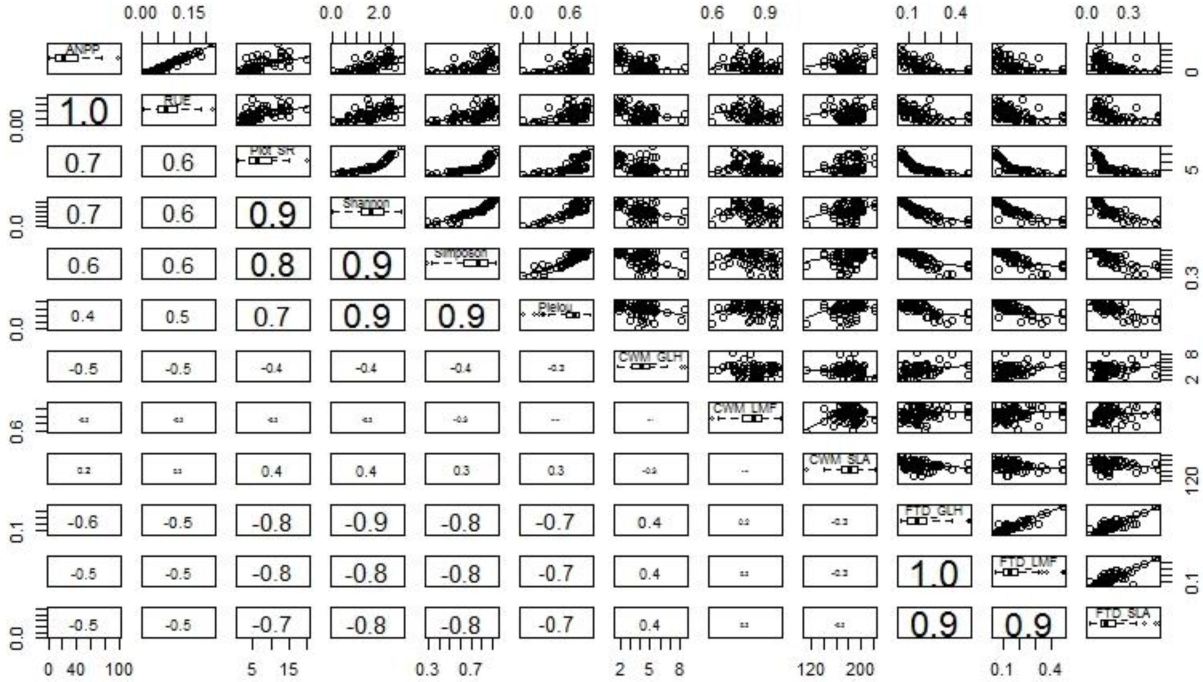

Figure S2 Correlation-Boxplot-Scatterplot matrix of community functionality (response variables) with / between abiotic variables}. Aboveground net primary productivity (ANPP) and precipitation use efficiency (PUE) were termed as community functions. Abiotic variables included longitude, latitude, Altitude, growing season precipitation (GSP), > 5 °C accumulated temperature (AccT), habitat moisture index (HMI, = GSP/AccT), soil organic carbon (SOC), total nitrogen (STN) and C:N ratio (SCNR). The upper panels show the pair-wise scatter plots with smoothers to visualize the patterns, the lower panels contain the Pearson correlation coefficients that is proportional to its estimated value and the diagonal panels show the box plot and the name of the variables which is on the *x*-axis below and above it, and on the *y*-axis left and right of it. The code was taken from the pairs help file in RStudio

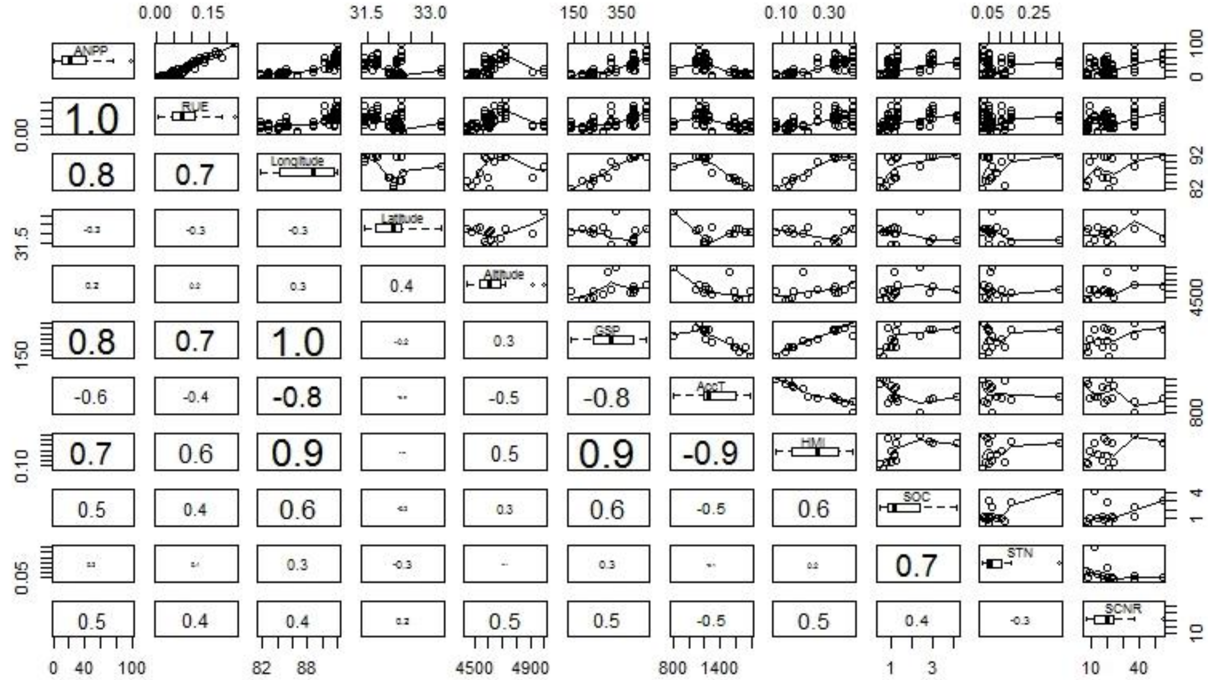

Table S2 Summary of the potential generalized additive models (GAM) for aboveground net primary productivity (ANPP), categorized into geospatial GAMs, edaphic GAMs, Climatic GAMs, and mixed GAMs. Abiotic variables included longitude (Long.), latitude (Lat.), altitude (Alt.), growing season precipitation (GSP), > 5°C accumulated temperature (AccT), habitat moisture index (HMI, = GSP/AccT), soil organic carbon (SOC), total nitrogen (STN) and C: N ratio (SCNR) were considered. Values of Adjusted R-square, Akaike Information Criterion (AIC) and Bayesian Information Criteria (BIC) for each model were provided.

GAM	Predictors	Est. *df*	Est. rank	*F*	*P*	Adj.R^2	AIC	BIC
Geospatial								
GAM1	Long.	1	1	25.240	< 0.001	0.801	561.67	592.69
	Lat.	5.999	6.671	2.154	0.084			
	Alt.	4.388	4.895	7.229	< 0.001			
GAM2	Long.	0.999	1	41.654	< 0.001	0.796	560.82	585.11
	Alt.	7.482	8.067	8.122	< 0.001			
Climatic								
GAM3	GSP	6.039	6.509	3.825	0.002	0.798	563.39	596.43
	AccT	5.217	5.680	2.342	0.058			
	HMI	1	1	6.348	0.014			
GAM4	GSP	7.350	7.788	9.787	< 0.001	0.784	565.70	591.37
	HMI	1.727	2.014	1.372	0.208			
GAM5	GSP	7.494	8.127	28.770	< 0.001	0.756	573.61	595.61
Edaphic								
GAM6	SOC	6.818	7.031	7.521	< 0.001	0.799	563.92	599.63
	STN	5.423	5.642	4.832	0.003			
	C/N	1.168	1.194	2.814	0.069			
GAM7	SOC	7.461	7.574	23.751	< 0.001	0.799	564.30	600.83
	STN	6.305	6.406	4.448	< 0.001			
Geospatial + Climatic + Edaphic								
GAM8	Long.	0.991	0.992	19.912	< 0.001	0.804	559.43	587.71
	Alt.	1	1	1.835	0.180			
	GSP	3.254	3.818	6.828	< 0.001			
	AccT	2.498	2.902	5.445	0.003			
	HMI	0.462	0.461	16.230	0.008			
	SOC	1	1	1.746	0.191			
	STN	1	1	4.940	0.030			
GAM9	Long.	1	1	9.054	0.004	0.801	561.18	591.51
	GSP	1.505	1.643	1.386	0.217			
	AccT	3.931	4.434	2.260	0.065			
	HMI	2.811	3.259	1.625	0.119			
	STN	1.838	2.020	2.022	0.137			
Geospatial + Climatic								
GAM10	GSP	5.773	6.488	15.519	< 0.001	0.798	561.71	586.69
	Alt.	3.439	3.984	4.634	0.003			
**GAM11**	**HIM**	**6.605**	**7.255**	**13.770**	**< 0.001**	**0.803**	**559.77**	**587.67**
	**Alt.**	**3.433**	**3.926**	**18.830**	**< 0.001**			

Table S3 Summary of the potential generalized additive models (GAM) for precipitation use efficiently (PUE), categorized into geospatial GAMs, edaphic GAMs, Climatic GAMs and mixed GAMs. Abiotic variables included longitude (Long.), latitude (Lat.), altitude (Alt.), growing season precipitation (GSP), > 5 °C accumulated temperature (AccT), habitat moisture index (HMI, = GSP/AccT), soil organic carbon (SOC), total nitrogen (STN) and C: N ratio (SCNR) were considered. Values of Adjusted R-square, Akaike Information Criterion (AIC) and Bayesian Information Criteria (BIC) for each model were provided.

GAM	Predictors	Est. *df*	Est. rank	*F*	*P*	Adj.R^2	AIC	BIC
Geospatial								
GAM1	Long.	4.198	4.768	5.208	< 0.001	0.671	-323.89	-296.64
	Lat.	2.194	2.534	0.526	0.600			
	Alt.	3.364	3.911	4.460	0.0046			
GAM2	Long.	4.433	5.058	6.244	< 0.001	0.671	-324.85	-299.72
	Alt.	4.411	5.101	3.745	0.0045			
Climatic								
GAM3	GSP	6.673	6.872	3.068	0.007	0.677	-322.57	-287.10
	AccT	5.632	5.959	1.971	0.07			
	HMI	1	1	3.617	0.06			
GAM4	GSP	7.494	7.923	6.823	< 0.001	0.646	-318.81	-192.61
	HMI	1.812	2.107	1.207	0.18			
GAM5	GSP	7.856	8.382	13.010	< 0.001	0.585	-308.28	-285.44
Edaphic								
GAM6	SOC	7.240	7.443	6.526	< 0.001	0.678	-322.64	-286.83
	STN	5.210	5.445	3.538	0.009			
	C/N	1	1	4.288	0.043			
GAM7	SOC	7.525	7.639	13.207	< 0.001	0.678	-322.32	-285.77
	STN	6.238	6.341	3.264	0.007			
Geospatial + Climatic + Edaphic								
GAM8	Long.	1	1	25.341	< 0.001	0.683	-325.94	-296.43
	Alt.	1	1	3.540	0.064			
	GSP	3.378	3.949	2.553	0.032			
	AccT	2.150	2.570	3.208	0.184			
	HMI	1	1	0.333	0.566			
	SOC	1	1	0.979	0.326			
	STN	1.206	1.327	2.568	0.065			
GAM9	Long.	3.051	3.520	5.009	0.002	0.686	-327.18	-299.03
	Alt	1.702	19.912	7.770	0.003			
	GSP	3.618	4.166	2.063	0.076			
	STN	1.776	2.001	2.498	0.090			
Geospatial + Climatic								
GAM10	GSP	6.589	7.300	6.277	< 0.001	0.676	-325.40	-298.91
	Alt.	2.842	3.290	6.307	< 0.001			
**GAM11**	**HIM**	**7.246**	**7.886**	**5.710**	**< 0.001**	**0.688**	**-327.74**	**-299.51**
	**Alt.**	**2.936**	**3.348**	**13.060**	**< 0.001**			

Figure S3 Comparisons of aboveground net primary productivity (ANPP, g m^{-2}), precipitation use efficiency (PUE, g m^{-2} mm^{-1}), species richness at plot level (Plot_SR), plant diversity indices of Shannon, Simponson and Pielou Evenness, community weighted means (CWM) and Functional trait divergence (FTD) for general leaf height (GLH), leaf mass fraction (LMF) and specific leaf area (SLA) among alpine grassland types, which are arranged from alpine meadow (AM) to steppe (AS) and desert-steppe (ADS) with a decreasing growing season precipitation (GSP) gradient across the Northern Tibetan Plateau. Different letters over/below the bars indicate significance at $P < 0.05$ (One-way ANOVA).

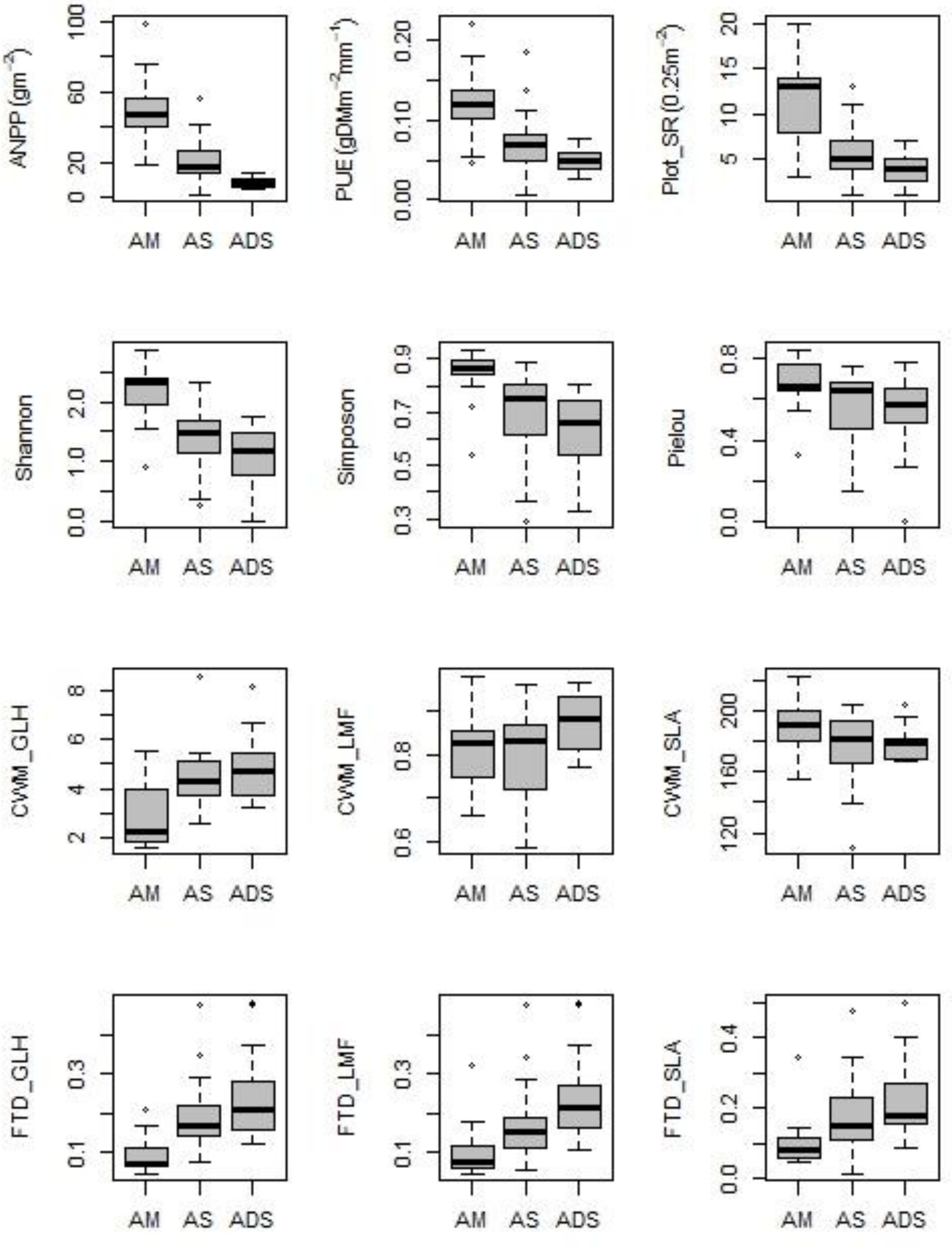

Figure S4 Structural equation models examining effects of environmental factors and biodiversity components on (a) aboveground net primary productivity (ANPP) and (b) precipitation use efficiency (PUE), respectively, at the regional scale across zonal alpine grassland types on the Northern Tibetan Plateau. Green and red arrows indicate significant positive and negative effects, respectively. Line width illustrates path strength. Values associated with arrows represent standardized path coefficients. Non-significant paths and variables were included in the full SEMs. Summaries of estimate and significance for regression, covariance were shown in Table S3. Plant species diversity (PSD) includes indices of richness, Shannon, Simpson, Pielou evenness at the plot level. Community weighted mean (CWM) and functional trait divergence (FTD) were calculated from general leaf height (GLH), specific leaf area (SLA), and leaf mass fraction (LMF). The methods for rescaling data were shown in Table S4.

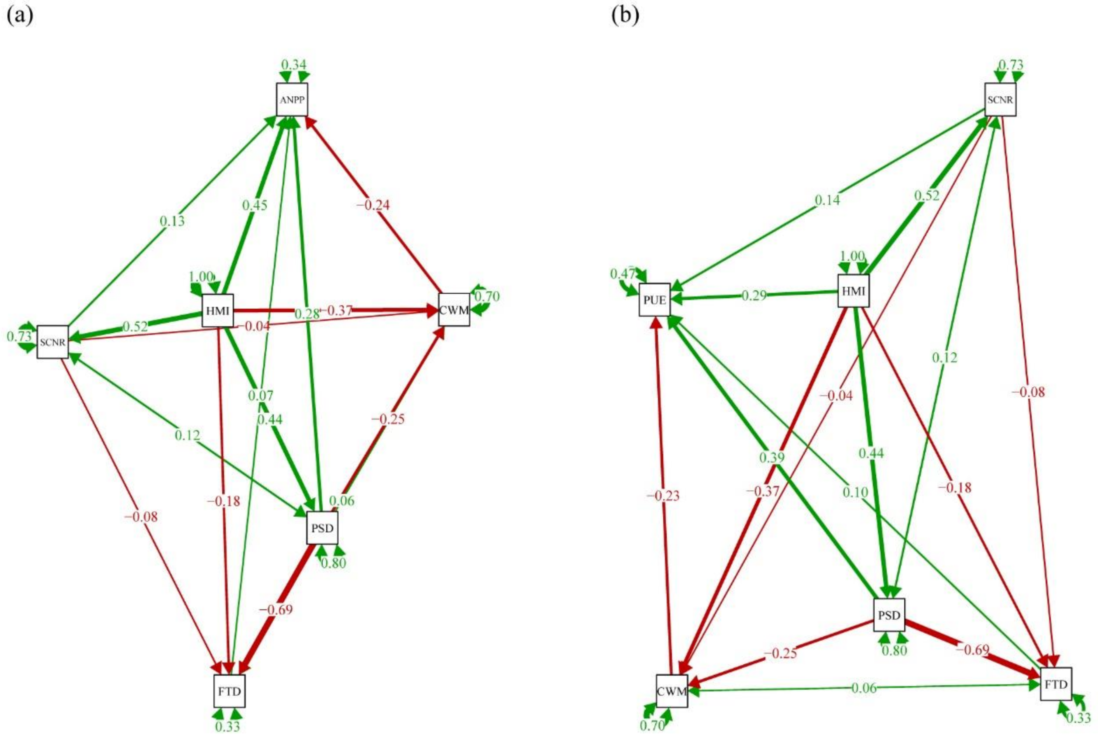

Table S4 Summaries of the full structure equation models (SEMs) for aboveground net primary productivity (ANPP) and precipitation use efficiency (PUE). Estimate and significance (*P* value) for regression and covariance were shown. R-square value for each dependent variable was also estimated. Habitat moisture index (HMI) that equals the ratio of growing season precipitation (GSP) to accumulated temperature when daily mean is over 5°C (AccT) and soil C: N ratio (SCNR) that combines carbon and nitrogen in soils were considered. Plant species diversity (PSD) includes indices of richness, Shannon, Simpson, Pielou evenness at the plot level. Community weighted mean (CWM) and functional trait divergence (FTD) were calculated from general leaf height (GLH), specific leaf area (SLA), and leaf mass fraction (LMF).
Values of Akaike Information Criterion (AIC) and Bayesian Information Criteria (BIC) for each model were provided.

SEM for ANPP			SEM for PUE		
Regressions:		Estimate	P(>|z|)	Estimate	P(>|z|)
ANPP ~	HMI	0.889	0.000	0.123	0.007
	PSD	0.574	0.011	0.170	0.003
	CWM	-0.992	0.003	-0.200	0.018
	**FTD**	**0.133**	**0.575**	**0.045**	**0.450**
	**SCNR**	**0.213**	**0.103**	**0.050**	**0.134**
CWM ~	HMI	-0.175	0.003	-0.175	0.002
	PSD	-0.122	0.020	-0.122	0.021
	**SCNR**	**-0.017**	**0.703**	**-0.017**	**0.705**
FTD ~	HMI	-0.173	0.034	-0.173	0.030
	PSD	-0.691	0.000	-0.691	0.000
	**SCNR**	**-0.065**	**0.305**	**-0.065**	**0.309**
PSD ~	HMI	0.429	0.000	0.429	0.000
SCNR~	HMI	0.616	0.000	0.616	0.000
Covariance					
CWM ~~	FTD	0.000	0.626	0.000	0.626
PSD ~~	SCNR	0.001	0.293	0.001	0.293
R-square	ANPP	0.649	R-square	PUE	0.529
	CWM	0.302		CWM	0.304
	FTD	0.664		FTD	0.672
	PSD	0.195		PSD	0.195
	SCNR	0.270		SCNR	0.270
Fitness	AIC	-930.347	Fitness	AIC	-1136.097
	BIC	-883.997		BIC	-1089.747

*Before model building, variables were recoded and rescaled as following

ANPP <-ANPP/100

PSD <-(Plot_SR/100+Shanno/10+Simposon+Pielou)/4

CWM <-(CWM_GLH/10+CWM_LMF+CWM_SLA/1000)/3

FTD <-(FTD_GLH+FTD_LMF+FTD_SLA)/3

SCNR<- SCNR/100
